# Management Strategies for Generalised Granuloma Annulare: A Systematic Review of Current and Emerging Therapies

**DOI:** 10.1111/ajd.14560

**Published:** 2025-06-30

**Authors:** Luca Bettolini, Vincenzo Maione, Andrea Carugno, Giorgio Stabile, Antonio Podo Brunetti, Zeno Fratton, Enzo Errichetti, Nicola Zerbinati, Franco Rongioletti, Piergiacomo Calzavara‐Pinton, Mariateresa Rossi, Stefano Bighetti

**Affiliations:** ^1^ Dermatology Department University of Brescia, ASST Spedali Civili di Brescia Brescia Italy; ^2^ Department of Medicine and Surgery University of Insubria Varese Italy; ^3^ Department of Clinical Dermatology Vita‐Salute San Raffaele University Milan Italy; ^4^ Unit of Dermatology IRCCS San Raffaele Hospital Milan Italy; ^5^ Department of Medicine, Institute of Dermatology University of Udine Udine Italy; ^6^ Department of Medicine and Technology University of Insubria Varese Italy

**Keywords:** disseminated granuloma annulare, generalised granuloma annulare, granuloma annulare, systematic review, therapy

## Abstract

Generalised Granuloma Annulare (GGA) is a chronic inflammatory skin disorder with no standard treatment. Since the last review in 2013, new treatments and varied responses have highlighted the need for an updated synthesis to guide clinical decisions. This systematic review aimed to evaluate the epidemiology, comorbidities and treatment outcomes in patients with GGA, synthesising evidence from published studies to provide insights into both conventional and emerging therapeutic strategies. A systematic literature search was conducted in CENTRAL, Embase and PubMed, following PRISMA guidelines. Studies published in English, French or Spanish up to January 15, 2024, were included. Data extraction focused on patient demographics, comorbidities, treatment regimens and therapeutic outcomes. A total of 689 patients were included. The mean age of patients was 52.8 years, with a female predominance (72.6%). Based on this systematic review, we propose a stepwise approach: first‐line treatment includes hydroxychloroquine and phototherapy (PUVA > UVA1 > nb‐UVB). Oral corticosteroids along with high‐potency topical steroids or calcineurin inhibitors may be used in extensive or rapidly progressive disease or as bridging therapy pending slower‐acting agents. Sulfones are second‐line, with oral retinoids (e.g., isotretinoin) as alternatives if contraindicated. For refractory cases, off‐label anti‐TNF‐α agents or JAK inhibitors are recommended, with methotrexate or cyclosporine as valid alternatives. This largest systematic review of GGA treatments offers an evidence‐based clinical framework. While steroids and phototherapy remain standard, emerging options like JAK inhibitors and biologics show promise for refractory cases. Tailored, multimodal strategies may improve outcomes, though further trials are needed to standardise guidelines.

Granuloma annulare (GA) is a benign inflammatory skin condition characterised by smooth, ring‐shaped plaques or papules with raised borders and central skin‐coloured or slightly depressed areas [1]. Localised GA (LGA) is the most common form, typically affecting the hands, feet and elbows. Generalised GA (GGA) is typically defined as involvement of the trunk and limbs, although it may also refer to more than 10 lesions across multiple anatomical areas, and it often presents as a chronic, recurrent and mildly pruritic condition. Less common variants include subcutaneous, perforating and patch types. GGA treatment is challenging due to unpredictable therapeutic responses. Currently, there are no consensus guidelines for the treatment of GGA. Treatment options include conventional therapies like steroids and phototherapy [2] as well as emerging approaches such as JAK inhibitors, biologics and combination strategies tailored to disease severity and patient comorbidities. Recently, a treatment algorithm for GGA was developed through a retrospective analysis of 18 patients [3]. This algorithm, developed from the latest systematic review of 2013 [4], along with expert opinion and clinical experience, tries to offer a structured approach to managing this challenging condition.

This systematic review aimed to summarise and evaluate the current treatment options for GGA, incorporating the latest evidence. It provides a detailed overview of available approaches and their effectiveness, offering insights to support clinical decision‐making and optimise patient care.

## 1 Materials and Methods

This systematic review, registered in PROSPERO (CRD42025632216), follows PRISMA guidelines and does not require ethical review or informed consent. A comprehensive search of CENTRAL, and PubMed was conducted using terms related to generalised granuloma annulare and treatment, focusing on studies in English, French and Spanish involving human subjects through January 15, 2025.

Two authors (L.B. and S.B.) independently screened titles and abstracts, resolving disagreements with a third author (V.M.). Given the rarity of GGA, we included studies with the following designs: case reports, case series, cross‐sectional studies and cohort studies. Conference abstracts and review articles were excluded. Additionally, studies that did not report therapies or outcome data were excluded from consideration.

**FIGURE 1:**
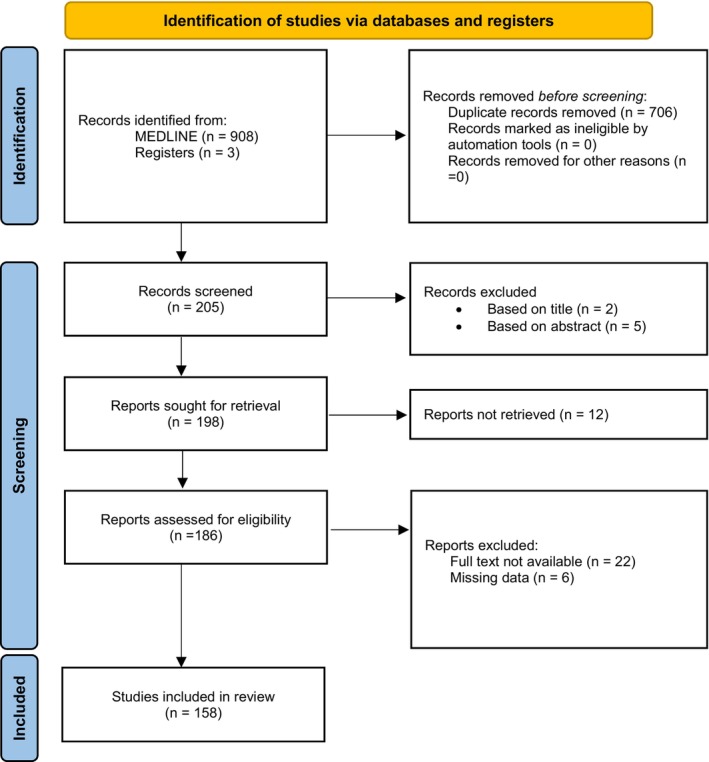
PRISMA flow diagram of study selection.

As illustrated in Figure 1, the search yielded 911 records. After the removal of 706 duplicates and initial screening of titles and abstracts, 186 potentially eligible studies were identified. Subsequently, after eligibility assessment, 158 full‐text articles were thoroughly reviewed and evaluated for inclusion in the analysis (Figure 1).

Two researchers (L.B. and S.B.) independently extracted data, including title, first author, publication year, study population, participant demographics, prior and current treatments with outcomes, side effects and comorbidities (Table 1, Tables S1 and S2) [1, 4, 5, 6, 7, 8, 9, 10, 11, 12, 13, 14, 15, 16, 17, 18, 19, 20, 21, 22, 23, 24, 25, 26, 27, 28, 29, 30, 31, 32, 33, 34, 35, 36, 37, 38, 39, 40, 41, 42, 43, 44, 45, 46, 47, 48, 49, 50], [S1–S110]. Discrepancies during screening, data extraction or bias assessment were resolved through discussion, with a third investigator (V.M.) providing adjudication if needed.

**TABLE 1:** Baseline characteristics.

Parameter	*N* = 689
Age at evaluation (y), weighted mean (range)	52.8^a^ (0.2–89)
Gender, *n* (%)	
Female	500 (72.7)
Male	188 (27.3)
Comorbidities	
Dysglycemia, *n* (%)	106 (15.4)
Type 2 Diabetes Mellitus	100 (14.5)
Type 1 Diabetes Mellitus	3 (0.4)
Prediabetes	2 (0.3)
Hyperglycemia	1 (0.1)
Thyroid Disease, *n* (%)	59 (8.6)
Hypothyroidism	50 (7.3)
Dyslipidemia, *n* (%)	78 (11.3)
Hypertension, *n* (%)	32 (4.6)
Neoplasia, *n* (%)	
Haematology	15 (2.2)
Breast	13 (1.9)
Others	15 (2.2)
Infectious diseases, *n* (%)	
HBV	4 (0.6)
HCV	4 (0.6)
HIV	4 (0.6)
Tuberculosis	1 (0.1)
Others, *n* (%)	
Psoriasis	5 (0.7)
BCG Vaccination/Bladder Cancer Therapy	3 (0.4)
COVID‐19 Vaccine	3 (0.4)

^a^ Based on 688 patients.

## 2 Results

Our pooled analysis included 689 patients with GGA (Table 1), with a weighted mean age of 52.8 years (based on 688 patients) and an age range of 0.2–89 years. Of these, 72.6% (*n* = 500) were female, and 27.4% (*n* = 189) were male.

Comorbidities included type 2 diabetes mellitus in 100 patients (14.5%), type 1 diabetes mellitus in 3 patients (0.4%), prediabetes in 2 patients (0.3%) and isolated hyperglycaemia in 1 patient (0.1%). Thyroid disease was reported in 59 patients (8.6%), predominantly hypothyroidism (50 cases, 7.3%). Dyslipidaemia affected 78 patients (11.3%), hypertension in 32 (4.6%), haematologic neoplasia in 15 (2.2%), breast cancer in 13 (1.9%) and other malignancies in 15 (2.2%). Psoriasis was documented in 5 patients (0.7%).

Infectious diseases included HBV, HCV and HIV in 4 patients each (0.6%), and tuberculosis in 1 patient (0.1%). Additionally, 3 patients (0.4%) received BCG vaccination or immunotherapy for bladder cancer, and another 3 (0.4%) reported receiving the COVID‐19 vaccine prior to lesion onset.

### 2.1 Prior Treatments

A total of 754 treatments were recorded, with steroids being the most common (46.69%), including topical (34.5%, *n* = 260), intralesional (5.7%, *n* = 43) and oral (6.5%, *n* = 49) administrations. Non‐corticosteroid topicals included calcineurin inhibitors (3.7%, *n* = 28), retinoids (0.7%, *n* = 5) and single cases of calcipotriol, dapsone, ruxolitinib and benvitimod. Physical therapies accounted for 9.6% (*n* = 72), with nb‐UVB (*n* = 28), PUVA/UVA1 (*n* = 27), laser (*n* = 2) and cryotherapy (*n* = 3).

Among non‐corticosteroid oral therapies, hydroxychloroquine (7.2%, *n* = 54) was most frequent, followed by dapsone (5.4%, *n* = 41), methotrexate (3.7%, *n* = 28), ROM (rifampicin, ofloxacin, minocycline) therapy (2%, *n* = 15), retinoids (2.5%, *n* = 19) and pentoxifylline (2.4%, *n* = 18). Less common treatments included antihistamines (0.8%, *n* = 6), cyclosporine (0.5%, *n* = 4) and tetracyclines (2%, *n* = 15). Rare options like vitamin E (0.8%, *n* = 6), adalimumab (0.9%, *n* = 7), apremilast, azathioprine, terbinafine (each 0.4%, *n* = 3) and single cases of naltrexone and mycophenolate mofetil (MMF) (0.1%) were also reported.

### 2.2 Current Therapies

#### 2.2.1 Corticosteroid Therapy

Topical steroids in monotherapy were administered to 96 patients. Among these, 17.7% (*n* = 17) achieved a complete response, 43.8% (*n* = 42) experienced a partial response, whereas 38.5% (*n* = 37) showed no response to treatment. Oral steroids were prescribed for 14 patients, achieving a complete response in 85.7% (*n* = 12), including two cases where topical steroids were used in combination. A partial response was observed in 14.3% (*n* = 2) of patients. Nine patients were treated with intralesional corticosteroids, with 33.3% (*n* = 3) achieving a complete response, 55.6% (*n* = 5) showing partial improvement and 11.1% (*n* = 1) experiencing no response.

#### 2.2.2 Non‐Corticosteroid Topical Therapies

Among non‐corticosteroid topical therapies, topical calcineurin inhibitors in monotherapy were the most frequently prescribed (*n* = 5). Of these, topical tacrolimus achieved a complete response in 2 cases (40%), while the remaining cases treated with topical tacrolimus and 1 case with topical pimecrolimus showed partial responses (60%). Topical JAK inhibitors (*n* = 4, including ruxolitinib and tofacitinib) achieved a complete response in one case (25%), while the remaining three cases (75%) showed no effectiveness. Finally, two patients received canary seed milk, achieving a complete response. Topical vitamin E demonstrated near‐complete clearance in one case, while topical anthralin showed partial effectiveness in two patients.

#### 2.2.3 Physical Modalities

A total of 196 physical treatments were recorded. Fifty‐six patients underwent nb‐UVB therapy, with 30.4% (*n* = 17) achieving a complete response, 37.5% (*n* = 21) a partial response, and 32.6% (*n* = 18) showing no response. For PUVA therapy, 83 patients were treated, of whom 49.4% (*n* = 41) achieved a complete response, 37.6% (*n* = 31) a partial response and 13.3% (*n* = 11) showed no response. Among 41 patients treated with UVA1 therapy, 34.2% (*n* = 14) achieved a complete response, 58.5% (*n* = 24) a partial response, and 7.3% (*n* = 3) showed no response. Two patients were treated with both nb‐UVB and PUVA therapy, resulting in a complete response in one patient and a partial response in the other. Additionally, three cases of methyl aminolevulinate‐PDT were reported, all achieving a complete response. Regarding laser therapy, pulsed dye laser was partially effective in eight patients, while excimer laser resulted in complete resolution in the treated lesions of three patients.

#### 2.2.4 Oral Retinoids

A total of 16 cases of oral retinoid treatment were recorded, including 2 with acitretin (12.5%), 1 with alitretinoin (6.3%), 2 with etretinate (12.5%) and 11 with isotretinoin (68.8%). Among these, 68.8% (*n* = 11) achieved a complete response, 18.8% (*n* = 3) had a partial response, and 12.5% (*n* = 2) showed no response.

#### 2.2.5 Sulfones

A total of 62 patients were treated with sulfones, including 51 with dapsone (82.3%) and 11 with sulfasalazine (17.7%). Among those treated with dapsone, 33.3% (*n* = 17) achieved complete remission, 25.5% (*n* = 13) showed a partial response, and 41.2% (*n* = 21) experienced no effect. Of the patients treated with sulfasalazine, 63.6% (*n* = 7) achieved complete remission, 18.2% (*n* = 2) showed a partial response, and 18.2% (*n* = 2) experienced no effect.

#### 2.2.6 Phosphodiesterase‐4 Inhibitor (PDE‐4i)

PDE‐4i (apremilast) were administered to 16 patients. Among them, 4 (25%) achieved a full resolution of their symptoms, 9 (56.3%) demonstrated a partial improvement, and 3 (18.8%) showed no significant response to the treatment.

#### 2.2.7 Anti‐Malarial Agents

A total of 85 patients were treated with antimalarial agents, including 77 (90.6%) who received hydroxychloroquine and 8 (9.4%) who received chloroquine. Among those treated with hydroxychloroquine, 44.2% (*n* = 34) achieved a complete response, 13% (*n* = 10) showed a partial response, and 42.9% (*n* = 33) experienced no improvement. In the chloroquine group, 75% (*n* = 6) achieved complete remission, 12.5% (*n* = 1) showed a partial response, and 12.5% (*n* = 1) experienced no improvement.

#### 2.2.8 Fumaric Acid Esters (FAEs)

FAEs were administered to 22 patients, with 12 receiving a combination of dimethyl fumarate and monoethyl fumarate (54.6%) and 10 treated with dimethyl fumarate alone (45.5%). Among all patients, 40.9% (*n* = 9) achieved complete remission, 50% (*n* = 11) showed partial improvement, and 9.1% (*n* = 2) experienced no response.

#### 2.2.9 Antimicrobial Drugs

A total of 45 antimicrobial treatments were recorded. Among 8 patients treated with doxycycline, 12.5% (*n* = 1) achieved a complete response, 25% (*n* = 2) showed partial improvement, and 62.5% (*n* = 5) experienced no response. Minocycline was administered to 11 patients, with 9.1% (*n* = 1) achieving a complete response, 36.36% (*n* = 4) showing partial improvement, and 54.5% (*n* = 6) experiencing no response. Notably, six patients treated with minocycline also received concomitant ofloxacin and rifampicin, with outcomes not separately reported. ROM therapy was prescribed to 11 patients, all of whom achieved complete clearance. Ten patients received colchicine, with 10% (*n* = 1) achieving a complete response, 50% (*n* = 5) showing partial improvement and 40% (*n* = 4) experiencing no response. Notably, one colchicine responder received concomitant treatment with dapsone. Rifampicin combined with ofloxacin and amoxicillin/clavulanate was used in one patient, resulting in a partial response, whereas amoxicillin/clavulanate alone was also associated with a partial response in one patient. Additionally, one patient treated with metronidazole achieved a complete response, and one patient treated with griseofulvin showed partial improvement. Clofazimine was administered to one patient, resulting in a partial response.

#### 2.2.10 Immunosuppressants

Thirty‐one patients were treated with immunosuppressants, including 25 with methotrexate (80.7%), 5 with cyclosporine (16.1%) and 1 with MMF (3.2%). Among those receiving methotrexate, 28% (*n* = 7) achieved a complete response, 28% (*n* = 7) showed partial improvement, and 44% (*n* = 11) experienced no response. For cyclosporine, 40% (*n* = 2) achieved complete remission, 20% (*n* = 1) showed partial improvement, and 40% (*n* = 2) experienced no response. The single patient treated with MMF achieved a complete response.

#### 2.2.11 Pentoxifylline

Pentoxifylline was administered to 34 patients, with 8.8% (*n* = 3) achieving a complete response, 29.4% (*n* = 10) showing partial improvement and 61.8% (*n* = 21) experiencing no response. It is worth noting that three of the responders received concomitant therapy with either dapsone, methotrexate or hydroxychloroquine, though the specific combinations with outcomes were not documented.

#### 2.2.12 Biological Therapies

Thirty biological therapies were recorded, including 25 anti‐TNF‐α agents (adalimumab [*n* = 20, 66.7%], infliximab [*n* = 3, 10%], certolizumab [*n* = 1, 3.3%] and golimumab [*n* = 1, 3.3%]), two dupilumab (6.7%), one efalizumab (withdrawn from the market) (3.3%) and two interleukin‐23 inhibitors (tildrakizumab, 6.7%). Among patients treated with anti‐TNF‐α therapies, 84% (*n* = 21) achieved a complete response, and 12% (*n* = 3) showed partial improvement. For dupilumab, 1 patient (50%) achieved a complete response, whereas the other showed partial improvement. The single patient treated with efalizumab achieved a complete response. Of the two patients treated with tildrakizumab, 1 (50%) showed partial improvement, and 1 (50%) experienced no response.

#### 2.2.13 Janus Kinase (JAK) Inhibitors

Twenty‐nine JAK inhibitors were prescribed, including 15 tofacitinib (51.7%), 10 upadacitinib (34.5%), 3 baricitinib (10.3%) and 1 abrocitinib (3.5%). Among these, 82.8% (*n* = 24) achieved a complete response, with 11 from tofacitinib, 9 from upadacitinib, 3 from baricitinib, and 1 from abrocitinib. The remaining 17.2% (*n* = 5) showed partial improvement.

#### 2.2.14 Oral Vitamin E

Four patients were treated with a combination of oral vitamin E and the 5‐lipoxygenase inhibitor zileuton, with three achieving a complete response and one showing partial improvement. Additionally, one patient received vitamin E in combination with tea tree oil, resulting in a partial response.

#### 2.2.15 Other Therapies

Thirteen patients were treated with potassium iodide, with one achieving a complete response, eight showing partial improvement and four experiencing no response. Two patients received hydroxyurea with a complete response. Defibrotide achieved a complete response in 1 patient.

#### 2.2.16 Treating Underlying Conditions

One patient achieved complete resolution following the discontinuation of pegylated interferon‐alpha for HCV infection. Similarly, three patients resolved after initiating treatment for HIV infection. Five patients experienced remission after addressing underlying conditions, including thyroid disorders, diabetes, chronic hepatitis C, chronic hepatitis B and tuberculosis infection. Five patients achieved remission after treating associated neoplasms, which included non‐Hodgkin lymphoma, concomitant ovarian and stomach neoplasia, cervical adenocarcinoma and chronic myelomonocytic leukaemia (CMML). One patient with Hodgkin lymphoma showed a partial response, while two with CMML and breast cancer did not achieve resolution. Additionally, one patient achieved resolution after the removal of surgical hardware (a stainless steel volar plate), and another with lesion onset following BCG vaccination experienced spontaneous complete remission.

#### 2.2.17 Combined Therapies

A total of 33 patients received combined therapy, defined as the use of at least two systemic therapies listed above, or at least one systemic therapy combined with one physical modality, or the combination of systemic or physical modalities with intralesional corticosteroids. Among these patients, 14 achieved complete resolution, 16 showed partial improvement, and 3 experienced no response.

## 3 Discussion

**FIGURE 2:**
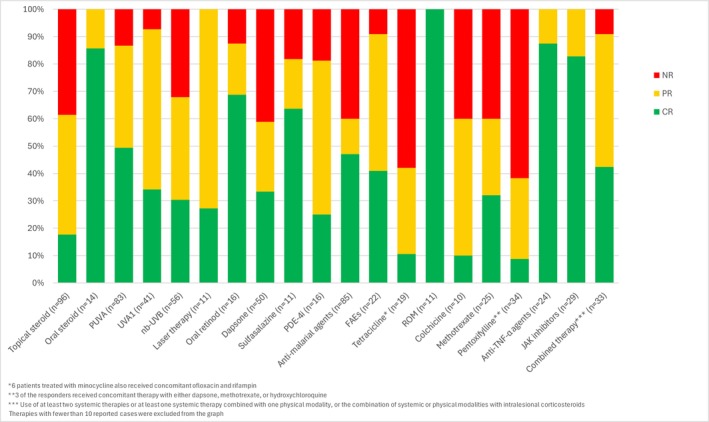
Treatment outcomes in patient with GGA: Results from the systematic review.

GGA presents a significant therapeutic challenge due to its chronic, recurrent nature and unpredictable response to available treatments. Our systematic review offers a comprehensive guide for clinicians in managing this refractory condition (Figure 2).

### 3.1 Corticosteroid Therapies

Topical steroids, despite their widespread use, demonstrated variable efficacy, with only 17.7% of patients achieving complete resolution. Intralesional corticosteroids yielded better outcomes, with a complete response observed in one‐third of cases. Oral steroids, in contrast, showed a higher efficacy profile, with 85.7% of patients achieving remission. Notably, most patients had previously undergone steroid treatment, both orally and topically. Although the reasons for discontinuation cannot be definitively determined, it is plausible that interruptions were due to lack of efficacy, relapse upon withdrawal or adverse events.

### 3.2 Non‐Corticosteroid Topical and Physical Therapies

Non‐corticosteroid therapies exhibited a diverse efficacy spectrum. Topical calcineurin inhibitors, achieved complete resolution in 40% of cases. Among physical modalities, phototherapy, particularly PUVA, emerged as a cornerstone treatment, with nearly half of the patients achieving complete resolution. UVA1 and nb‐UVB also demonstrated notable efficacy, albeit with slightly lower response rates (34.2% and 30.4%, respectively). Laser‐based treatments, were effective in a subset of patients, supporting their use in refractory localised lesions.

### 3.3 Conventional Systemic Therapies

Retinoids also played a role in treatment, achieving 68.75% of complete response in a small cohort. Antimalarial agents, predominantly hydroxychloroquine, were widely prescribed but showed mixed results, with complete remission achieved in less than half of the cases (*n* = 40/85). Similarly, antimicrobial drugs, such as doxycycline, minocycline and ROM therapy, demonstrated variable outcomes, with ROM therapy showing the highest rates of complete response among the group. However, as a prior treatment, ROM therapy resulted in 15 failures compared to 11 successes. The available data may be influenced by publication bias and likely underreport treatment failures, warranting further studies to establish its true efficacy. Sulfones, particularly dapsone, provided moderate efficacy, with approximately one‐third of patients achieving complete response. Sulfasalazine demonstrated promising efficacy in a small number of cases, highlighting the need for further research to better understand its potential. The utility of FAEs and PDE‐4i was limited by variable outcomes, making it difficult to draw definitive conclusions about their effectiveness. Among oral immunosuppressants, methotrexate and cyclosporine were the most frequently used agents. Methotrexate achieved complete resolution in 28% of cases, while cyclosporine showed higher efficacy, though its use was significant lower.

### 3.4 Biologic and Targeted Therapies

Biological therapies, particularly anti‐TNF‐α agents, demonstrated robust efficacy, with over 80% of patients achieving complete or partial responses. Similarly, JAK inhibitors, such as tofacitinib and upadacitinib, achieved high rates of complete resolution, underscoring their potential as emerging therapeutic options.

### 3.5 Comorbidities and Combination Approaches

Treatment of underlying conditions was associated with significant clinical improvement in a subset of patients. Resolution was observed in cases addressing HIV infection, thyroid disorders, diabetes, chronic viral hepatitis, tuberculosis and malignancies. This highlights the importance of identifying and managing comorbidities or triggering factors in patients with GGA. Combined therapeutic approaches, integrating systemic therapies with physical modalities or intralesional corticosteroids demonstrated notable efficacy. Among 33 patients receiving combination treatments, nearly half achieved complete resolution, suggesting that tailored multimodal regimens may optimise outcomes in refractory cases.

### 3.6 Pathogenesis and Therapeutic Targets

The pathogenesis of GGA is not yet fully elucidated, but current evidence suggests a delayed‐type hypersensitivity reaction mediated by a Th1‐dominant immune response [S111]. Elevated levels of TNF‐α, interferon‐gamma, and interleukin‐2 have been identified in lesional skin, implicating T‐cell‐driven granulomatous inflammation^111^. These immunological findings provide a plausible mechanistic basis for the observed efficacy of immunomodulatory therapies, such as TNF‐α inhibitors, JAK inhibitors and methotrexate, which disrupt pro‐inflammatory cytokine signalling. Similarly, the benefit of phototherapy—particularly PUVA and UVA1—may be explained by its capacity to induce T‐cell apoptosis, modulate antigen‐presenting cells and downregulate cytokine expression. Retinoids, such as acitretin, may exert therapeutic effects through the regulation of keratinocyte differentiation, anti‐inflammatory activity and inhibition of granuloma formation, although direct mechanistic evidence in GGA is limited. In contrast, the mechanisms underlying the reported efficacy of some less conventional treatments remain uncertain and are not yet supported by established pathophysiological models. Outcomes described in isolated case reports should be interpreted with appropriate caution, as spontaneous remission or placebo effects cannot be excluded. Further research is warranted to better elucidate the immunopathogenesis of GGA and to ensure that therapeutic strategies are grounded in a clear mechanistic rationale.

### 3.7 Management Algorithm

**FIGURE 3:**
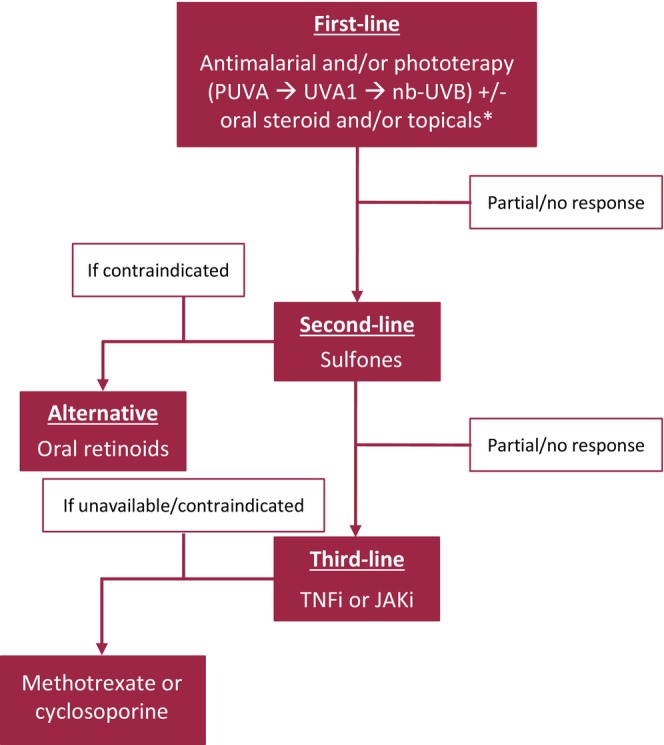
Management strategies for generalised granuloma annulare. *High‐potency topical steroids or topical calcineurin inhibitors. JAKi, Janus Kinase inhibitors; TNFi, tumour necrosis factor inhibitors.

Brerk‐Krauss and colleagues recently proposed treatment algorithm for GGA [3]. Antimalarials were the first‐line therapy, with nb‐UVB phototherapy offered as an alternative or adjunct for photoprotected lesions with patch morphology, excluding photodistributed GGA. Topical anti‐inflammatories and intralesional triamcinolone were used for localised, symptomatic lesions. Adjunct therapies like doxycycline and pentoxifylline were considered for partial responders or as low‐risk options. Second‐line treatment involved TNF inhibitors for refractory cases, while dapsone, methotrexate, and acitretin were reserved for selected patients due to lower efficacy and higher side effects. Based on the findings of this systematic review, and incorporating additional clinical considerations—including safety profiles, treatment accessibility and expert experience—we propose the following stepwise escalation strategy (Figure 3): initiating treatment with hydroxychloroquine and phototherapy (if available, prioritising PUVA over UVA1, and UVA1 over nb‐UVB). Oral corticosteroids along with high‐potency topical corticosteroids or topical calcineurin inhibitors can be added in cases of extensive or rapidly progressive disease, or as a bridging therapy to achieve rapid symptom control while awaiting the effect of slower‐acting agents. In a retrospective study on UVA1 [2], the response rates for GGA were consistent with our review, achieving a clear or almost clear response in 22 out of 54 patients (40.7%) and 14 out of 41 patients (34.1%), respectively. Given the poor response rates observed with pentoxifylline and doxycycline, we suggest sulfones as second‐line therapies. In cases where this option is contraindicated, oral retinoid (e.g., isotretinoin) may be considered. This aligns with findings from the retrospective analysis by Brerk‐Krauss and colleagues, where none of the patients responded to pentoxifylline or doxycycline [3]. For recalcitrant GGA, we recommend considering the off‐label use of anti‐TNF‐α agents and JAK inhibitors. When these are unavailable, immunosuppressants such as methotrexate or cyclosporine can be added as alternative options. Given the limited quality of available evidence and the typically indolent course of GGA, observation without active treatment may be a reasonable option in selected cases, particularly in patients with mild or asymptomatic disease, where clinical burden is low and shared decision‐making supports a conservative approach.

## 4 Limitations

The heterogeneity of included studies, variability in outcome measures and the absence of standardised treatment guidelines remain significant limitations. Importantly, all studies were either retrospective or consisted of case series/studies, with no randomised controlled trials or head‐to‐head comparative studies available. Additionally, the lack of long‐term follow‐up data precludes assessment of sustained remission rates. Future research should focus on randomised controlled trials to establish evidence‐based guidelines, explore the efficacy of emerging therapies and evaluate combination approaches.

## 5 Conclusions

This systematic review highlights the complexity of managing GGA and underscores the need for individualised, multimodal treatment strategies. While conventional therapies such as steroids and phototherapy remain mainstays, emerging options like JAK inhibitors and biological agents offer promising avenues for refractory cases. Addressing underlying conditions and utilising combination therapies further enhances treatment outcomes, paving the way for more effective and personalised management of this challenging condition. Future studies are warranted to confirm these observations and support evidence‐based management strategies.

## Author Contributions

**Luca Bettolini:** conceptualization, data curation, investigation, project administration, resources, writing – original draft, writing – review and editing. **Vincenzo Maione:** conceptualization, data curation, project administration, writing – review and editing. **Andrea Carugno:** supervision, writing – original draft. **Giorgio Stabile:** data curation, resources. **Antonio Podo Brunetti:** formal analysis, methodology, validation. **Zeno Fratton:** investigation, resources, visualization. **Enzo Errichetti:** supervision, validation. **Nicola Zerbinati:** supervision, resources. **Franco Rongioletti:** supervision. **Piergiacomo Calzavara‐Pinton:** supervision, validation. **Mariateresa Rossi:** methodology, writing – original draft, visualization. **Stefano Bighetti:** conceptualization, data curation, investigation, project administration, resources, writing – original draft.

## Ethics Statement

Protocol registered in PROSPERO (CRD42025632216).

## Conflicts of Interest

The authors declare no conflicts of interest.

## Supporting information


**Table S1.** Prior treatments.

**Table S2.** Study characteristics.

Data S1.

## Data Availability

The data that support the findings of this study are available from the corresponding author upon reasonable request.
