# Supplementary material for: Management Strategies for Generalised Granuloma Annulare: A Systematic Review of Current and Emerging Therapies
Source: Australas J Dermatol. 2025 Jun 30;66(6):329–37. doi: 10.1111/ajd.14560 (PMC12418146; doi:10.1111/ajd.14560)
Supplement: Supplementary file 1 — Table S1. Prior treatments. [file AJD-66-329-s002.docx]

Supplementary table 1. Prior treatments.

| Prior treatment | N = 754 |
| --- | --- |
| Steroids, n (%) | 352 (46.7) |
| Topical | 260 (34.5) |
| Intralesional | 43 (5.7) |
| Oral | 49 (6.5) |
| Non-Steroidal Topicals, n (%) | 39 (5.2) |
| Calcineurin Inhibitors | 28 (3.7) |
| Retinoids | 5 (0.7) |
| Calcipotriol | 2 (0.3) |
| Dapsone | 1 (0.1) |
| Ruxolitinib | 1 (0.1) |
| Benvitimod | 1 (0.1) |
| Physical Therapies, n (%) | 72 (9.6) |
| nb-UVB | 28 (3.7) |
| PUVA/UVA1 | 27 (3.6) |
| Laser Therapy | 2 (0.3) |
| Cryotherapy | 3 (0.5) |
| Oral Retinoids, n (%) | 19 (2.5) |
| Hydroxychloroquine, n (%) | 54 (7.2) |
| Dapsone, n (%) | 41 (5.4) |
| Immunosuppressants, n (%) | 54 (7.2) |
| Methotrexate | 28 (3.7) |
| Cyclosporine | 4 (0.5) |
| Azathioprine | 3 (0.4) |
| Mycophenolate Mofetil | 1 (0.1) |
| Antimicrobial drugs, n (%) |  |
| Rifampin, ofloxacin, minocycline | 15 (2) |
| Tetracyclines | 15 (2) |
| Terbinafine | 3 (0.4) |
| Pentoxifylline | 18 (2.4) |
| Adalimumab | 7 (0.9) |
| Others, n (%) |  |
| Antihistamines | 6 (0.8) |
| Clofazimine | 3 (0.4) |
| Vitamin E | 6 (0.8) |
| Colchicine | 3 (0.4) |
| Potassium Iodide | 3 (0.4) |
| Apremilast | 3 (0.4) |
| Naltrexone | 1 (0.1) |
