# Supplementary material for: Management Strategies for Generalised Granuloma Annulare: A Systematic Review of Current and Emerging Therapies
Source: Australas J Dermatol. 2025 Jun 30;66(6):329–37. doi: 10.1111/ajd.14560 (PMC12418146; doi:10.1111/ajd.14560)
Supplement: Supplementary file 2 — Table S2. Study characteristics. [file AJD-66-329-s001.docx]

Supplementary table 2. Study characteristics

| **Authors (year)** | **Study Design** | **Patients number** | **Treatments, clinical response (number)** | **Side Effects** | **Treatment Duration** |
| --- | --- | --- | --- | --- | --- |
| Dev et al. (2025) | Retrospective Study | 15 | Tofacitinib CR (11); PR (4) | Hyperlipidemia (2) | Mean (DS): 5.9 (2.4) months |
| Berk-Krauss et al. (2024) | Retrospective Study | 18 | nb-UVB CR (1); PR (1); NR (3)  HCQ CR (1); PR (5); NR (7) Topical ruxolitinib/tofacitinib NR (3) Topical steroid NR (1) Chloroquine CR (1); PR (1); NR (1)  ILK PR (2); NR (1) PTX NR (4) DOX NR (5) Dapsone NR (3) MTX NR (2) Acitretin NR (1) ADA CR (3); PR (2) Certolizumab CR (1) Infliximab CR (1) | NA | NA |
| Chen et al. (2024) | Case Report | 1 | UPA CR (1) | None Reported | 14 weeks |
| Coican et al. (2024) | Case Report | 2 | UPA CR (2) | Mild headaches during the first few weeks (1) | 6 months (1) (ongoing); 2 months (1) (ongoing) |
| De Greef et al. (2024) | Case Report | 1 | UPA CR (1) | Hypercholesterolemia | 10 months |
| Jonathan et al. (2024) | Retrospective Study | 42 | Colchicine* CR (1); PR (5); NR (4) PTX** CR (1); PR (6); NR (15) Mynocicline*** CR (1); PR (4); NR (5)   *One colchicine responder received concomitant treatment with dapsone  ** Three pentoxifylline responders received concomitant therapy with either dapsone, methotrexate, or hydroxychloroquine.  *** Six minocycline patients received concomitant ofloxacin and rifampin  Patients treated with monotherapy: colchicine: 8; PTX: 16; mynocicline: 4 | Gastrointestinal disturbances: Colchicine (2)  Pentoxifylline (3) Dizziness: Pentoxifylline: (1) | Colchicine: 4.8 months (mean); pentoxifylline: 13.3 months (mean); minocycline: 6.4 months (mean) |
| Michels et al. (2024) | Case Report | 1 | Abrocitinib CR (1) | Mild nausea, herpes labialis | 5 months |
| Trees et al. (2024) | Case Report | 1 | UPA CR (1) |  | 2 months (ongoing) |
| Zheng et al. (2024) | Case Series | 3 | UPA CR (2); PR (1) | Nonpainful oral lesion, respiratory infections (strep throat and COVID-19), evening bone pain, and abdominal cramps (1); acne (1) | 8 months (1) (ongoing); 13 months (1) (ongoing); not mentioned (ongoing) |
| Tsai et al. (2024) | Case Report | 1 | nb-UVB + topical steroid NR (1) | Severe HIV-associated aepsis leading to fatal outcome | NA |
| Horoub et al. (2024) | Case Report | 1 | ADA + MTX + ILK CR (1) | Fatigue | 9 months (ongoing) |
| Tahir et al. (2024) | Case Report | 1 | Oral steroid + topical steroid CR (1) | None reported | 3 months |
| McIntyre et al. (2024) | Case Report | 1 | minocycline + ILK + topical steroid PR (1) minocycline + i.m. steroid PR (1) MTX PR (1) | Gait instability: mynocicline | MTX: 2.5 months |
| Lee et al. (2024) | Case Report | 1 | Oral metronidazole + topical steroid CR (1) | None reported | 10 days |
| Cojocaru et al. (2024) | Case Report | 1 | Isotretinoin CR (1) Oral steroid CR (1) | None reported | 3 months |
| Gabutti et al. (2023) | Case Report | 1 | Dimethylfumarate CR (2) | Mild lymphocytopenia | 3 years |
| Joshi et al. (2023) | Case Series | 8 | Apremilast CR (3); PR (2); NR (3) | Diarrhea (1) | Mean (DS): 9.62 (6.46) months |
| Kapetanovic et al. (2023) | Case Report | 1 | Isoniazid CR (1) | None reported | 6 months |
| Park et al. (2023) | Case Series | 2 | Canary seed milk CR (2) | None reported | 2 months (1); 3 months (1) |
| Song et al. (2023) | Case Report | 1 | DUPI PR (1) | None reported | 16 weeks |
| Lansang et al. (2023) | Case Report | 1 | MMF CR (1) | None reported | NA |
| Wang et al. (2023) | Case Report | 1 | Oral steroid + topical steroid CR (1) | None reported | 2 months |
| Calado et al. (2023) | Case Report | 1 | PUVA CR (1) | None reported | NA |
| Youh et al. (2023) | Case Report | 1 | Topical steroid PR (1) | None reported | NA |
| Slater et al. (2023) | Case Report | 1 | UPA CR (1) | None reported | 1 month (ongoing) |
| Paganini et al. (2023) | Case Report | 1 | DUPI CR (1) | None reported | 6 months (ongoing) |
| Kim et al. (2023) | Case Series | 2 | BARI CR (2) | None reported | 5 months (ongoing) (1); 2 months (ongoing) (1) |
| Connor et al. (2023) | Case Report | 1 | HCQ NR (1) ADA CR (1) | None reported | 5 months (ongoing) |
| Al Ameer et al. (2023) | Case Report | 1 | nb-UVB + oral antihistamine CR (1) | None reported | 3 months (ongoing) |
| Awad et al. (2022) | Case Report | 1 | Tildrakizumab PR (1) | None reported | NA |
| Chumsaengsri et al. (2022) | Case Report | 1 | Griseofulvin PR (1) | None reported | 2 months |
| Colwell et al. (2022) | Case Report | 1 | Oral vitamin E + TTO PR (1) | None reported | Oral vitamin E: 3 weeks. Topical TTO: NA |
| Hirin et al. (2022) | Retrospective Study | 26 | HCQ CR (2); NR (11) HCQ + topical steroid CR (1); PR (1); NR (7) HCQ + calcipotriol CR (1) HCQ + dapsone CR (1) HCQ + PTX + phototerapy PR (1) HCQ + PTX NR (1) | Photosensitivity (1); gastrointestinal upset (2); pruritus (1); hair loss (2); fatigue (1); and diffuse rash (1) | Mean: 10 months |
| Hrin et al. (2022) | Retrospective Study | 15 | MTX CR (2); PR (1); NR (4) MTX + topical tacrolimus and steroid CR (1); PR (1) MTX + oral and topical steroid CR (1); NR (1) MTX + ILK + topical steroid PR (1) MTX + acitretin + oral steroid PR (1) MTX + PTX + topical steroid NR (1) MTX + topical steroid NR (1) | Stomatitis (2); alopecia (1); fatigue (3) patients; elevated ALT levels (1) | Mean 16months |
| Hrin et al. (2022) | Retrospective Study | 26 | Dapsone CR (1); PR (5); NR (11) Dapsone + topical tacrolimus and steroid PR (1) Dapsone + phototerapy PR (2) Dapsone + ROM + PTX + topical steroid PR (1) Dapsone + topical steroid PR (3); NR (1) Dapsone + colchicine PR (1) | Subclinical myelosuppression (8); nausea (2); urinary tract infection (2) | Mean: 9.8 months |
| Lee at al. (2022) | Case Report | 1 | Alitretinoin CR (1) | None reported | 15 months |
| Mizawa et al. (2022) | Case Series | 2 | Excimer laser CR (2) | None reported | 3 months (8 sessions) (1); 9 months (9 sessions) (1) |
| Yan et al. (2022) | Case Report | 1 | BARI CR (1) | None reported | 5 months |
| Nguyen et al. (2022) | Case Report | 1 | ILK + topical steroid PR (1) HCQ + topical steroid PR (1) | None reported | 2 months (ongoing) |
| Cammarata et al. (2021) | Case Report | 1 | nb-UVB + topical tacrolimus CR (1) | None reported | 7 weeks |
| Dopytalska et al. (2021) | Case Report | 1 | Golimumab CR (1) | None reported | 2 years |
| Eid et al. (2021) | Case Report | 1 | DOX PR (1) | None reported | 2 months |
| Song (2021) | Case Report | 1 | Tildrakizumab NR (1) | None reported | 7 months |
| Hansel et al. (2021) | Case Series | 2 | Apremilast PR (2) | None reported | 13 months (ongoing) (1); 12 months (ongoing) (1) |
| Holland et al. (2021) | Case Series | 5 | PTX + oral vitamin E PR (4); NR (1) | None reported | 3 months |
| Sondermann et al. (2021) | Case Report | 1 | UPA CR (1)* *MTX was associated for one month then tapered | None reported | 4 months |
| Lam et al. (2021) | Retrospective Study | 28 | Topical steroid NR (28) nb-UVB NR (13) HCQ CR (13); NR (3) ADA CR (3) | None reported | 6 months |
| Yang et al. (2021) | Case Series | 13 | Sulfasalazine CR (7); PR (2); NR (2) Sulfasalazine + HCQ CR (1) Sulfasalazine + PTX + oral CS PR (1) | Neutropenia/gastrointestinal distress and elevated liver function tests (2) | NA |
| Megna et al. (2020) | Case Report | 1 | HCQ CR (1) | None reported | 8 weeks |
| Nordmann et al. (2020) | Retrospective Study | 61 | Topical steroid CR (6); PR (38); NR (5) UVA1 CR (3); PR (13); NR (1) Potassium iodatum PR (5); NR (2) PUVA CR (3); PR (7); NR (1) nb-UVB PR (7); NR (1) ILK CR (3); PR (2) HCQ CR (1); PR (3) | None reported | NA |
| King et al. (2020) | Case Report | 1 | GA lesions completely disappeared after treating the underlying neoplasm (1) | None reported | NA |
| Durgin et al. (2020) | Case Report | 1 | HCQ + topical tacrolimus NR (1) Dapsone NR (1) Topical tofacitinib CR (1) | None reported | 2 months |
| Blum et al. (2019) | Case Series | 2 | Apremilast PR (2) | None reported | 18 months (ongoing) (1); 4 months (ongoing) |
| Clapé et al. (2019) | Case Report | 1 | GA lesions did not disappear after treating the underlying neoplasm (1) | None reported | 14 months |
| Torisu et al. (2019) | Case Report | 1 | Oral steroid CR (1) | NA | NA |
| Xu et al. (2019) | Case Report | 1 | HCQ CR (1) | None reported | 6 months |
| Bishnoi et al. (2019) | Case Series | 4 | Apremilast CR (1); PR (3) | Mild diarrhea (1); myalgia (2); nausea (1) | NA |
| Chandan et al. (2018) | Case Series | 2 | Rifampicin + ofloxacin + amoxicillin/clavulate PR (1) amoxicillin/clavulate PR (1) PTX + DOX PR (1) | None reported | NA |
| Naka et al. (2018) | Retrospective Study | 11 | MTX CR (3); PR (4); NR (4) | Diarrhea (1); hair loss (1); elevated liver enzymes (1) | mean of 2.5 years for responsive patients; mean of 6 months for non-responsive patients |
| Nambiar et al. (2017) | Case Report | 1 | PTX CR (1) | None reported | 6 months |
| Chen et al. (2016) | Case Report | 1 | Removal of surgical hardware CR (1) | None reported | NA |
| Cozzani et al. (2016) | Case Report | 1 | Colchicine CR (1) | Gastritis (1) | 2 years (ongoing) |
| Mikami et al. (2016) | Case Report | 1 | nb-UVB PR (1) nb-UVB + GA lesions completely disappeared after treating the underlying disease CR (1) | None reported | 16 months |
| Pavlovsky et al. (2016) | Retrospective Study | 13 | nb-UVB CR (3); PR (10) | None reported | NA |
| Yong et al. (2016) | Case Series | 2 | nb-UVB CR (2) | None reported | 6 months |
| Errichetti et al. (2016) | Case Report | 1 | nb-UVB CR (1) | None reported | 3 months |
| Min et al. (2016) | Prospective Study | 7 | ADA CR (6); PR (1) | Alopecia areata (1) | Mean: 31.86 months |
| Bala et al. (2016) | Case Series | 2 | nb-UVB + acitretin CR (2) Acitretin CR (1) | None reported | NA |
| Cunningham et al. (2015) | Retrospective Study | 20 | PUVA CR (5); PR (3); NR (4) CR/PR (7) at 6-months FU; CR/PR (5) at 12-months FU nb-UVB CR (4); PR (2)  CR/PR (3) at 6-months FU; CR/PR (2) at 12-months FU PUVA + nb-UVB CR (1); PR (1) | PUVA: Itch (1) NBUVB: itch and paraesthesia (1) | NA |
| Garg et al. (2015) | Prospective Study | 5 | ROM CR (5) | None reported | 4 to 8 months |
| Mahmood et al. (2015) | Case Report | 1 | ADA CR (1) | None reported | 12 months |
| Solano-López et al. (2014) | Case Report | 1 | nb-UVB CR (1) | None reported | 2.5 months |
| Acharya (2013) | Case Series | 2 | Dimethylfumarate and monoethylfumarate PR (2) | Mild and transitory side effects: itching, flushing, diarrhea, stomach pains (1); Temporary leukopenia and lymphopenia (1) | 3 years (1); 1 year (1) |
| Pătraşcu et al. (2013) | Retrospective Study | 8 | PTX + oral antihistamine + topical steroid* PTX + HCQ + oral antihistamine + topical steroid PR (2) PTX + oral antihistamine + topical steroid and calcipotriol* PTX + oral antihistamine + topical tacrolimus*  * PR in 4 out of 6 total cases | None reported | 3 months |
| Bégon et al. (2012) | Case Report | 1 | HCQ NR (1) PUVA NR (1) Minocycline NR (1) MTX CR (1) | None reported | 12 months with MTX |
| Andreu-Barasoain et al. (2012) | Case Report | 1 | Chloroquine CR (1) | None reported | 6 years |
| Bronfenbrener et al. (2012) | Case Report | 1 | Excimer laser therapy CR (1) | None reported | 15 treatment session |
| Browne et al. (2011) | Retrospective Study | 33 | PUVA CR (50%); PR (41%); NR (9%) CR (15) at 6-months FU; CR (6) at 12-months FU; CR (3) at 24-months | Erythema (5); HSV infection (1); polymorphic light eruption (5) | Median follow-up of 7 years (IQR 5–10 years) |
| Dornelles et al. (2011) | Case Report | 1 | Dapsone PR (1) | None reported | 3 months |
| Jantke et al. (2011) | Case Series | 2 | Anthralin PR (2) | Severe local skin irritation (2) | NA |
| Passeron et al. (2011) | Retrospective Study | 8 | 595-nm pulsed dye laser PR (8) | Transient hyperpigmentation, crusting, post-inflammatory hyperpigmentation | 8 to 18 weeks (2 to 3 sessions at 4–6-week intervals) |
| Wollina et al. (2011) | Retrospective Study | 23 | bath PUVA + dimethyl fumarate and monoethyl fumarate + topical steroid CR (3); PR (3) PUVA + dimethyl fumarate and monoethyl fumarate + topical steroid CR (3) bath PUVA + topical steroid CR (4); PR (4) Topical steroid PR (2) PUVA + topical steroid and calcineurin inhibitor PR (1); NR (1) PUVA PR (1) bath PUVA + dimethyl fumarate and monoethyl fumarate CR (1) | FAE-related adverse effects: gastrointestinal symptoms (abdominal pain, diarrhea, nausea) (2); mild, temporary increase in liver enzymes (2); and lymphopenia (2) | FAE treatment lasted for 8–12 weeks. PUVA treatment included multiple sessions depending on individual tolerability and response. |
| Bhushan et al. (2011) | Case Report | 1 | GA lesions mostly disappeared after treating the underlying disease | None reported | NA |
| Nagase et al. (2011) | Case Report | 1 | Spontaneous complete remission. | None reported | NA |
| Torres et al. (2011) | Case Report | 1 | ADA CR (1) | None reported | NA |
| Mazzatenta et al. (2010) | Case Series | 3 | Allopurinol CR (2); PR (1) | None reported | 2 to 6 months |
| Miličić et al. (2010) | Case Report | 1 | Oral steroid CR (1) | None reported | 2 monhts |
| Murdaca et al. (2010) | Case Report | 1 | Infliximab CR (1) | None reported | 10 months |
| Ine et al. (2010) | Case Report | 1 | Nb-UVB CR (1) | None reported | 2 months |
| Werchau et al. (2010) | Case Report | 1 | ADA CR (1) | None reported | NA |
| Marcus et al 2009) | Case Series | 6 | ROM CR (6) | Mild insomnia (1); discoloration of body fluids | 3-5 months |
| Piaserico et al. (2009) | Case Series | 3 | MAL-PDT CR (3) | Mild pain during radiation | 3 sessions (2); 5 sessions (1) |
| Weber et al. (2009) | Retrospective Case Series | 8 | Dimethyl fumarate and monoethyl fumarate CR (1); PR (5); NR (1); NE (1) | nausea, diarrhea (2); transient leucopenia (6); eosinophilia (1) | From 1 to 18 months. |
| Aşkin et al. (2009) | Case Report | 1 | Topical steroid CR (1) | None reported | NA |
| Duarte et al. (2009) | Case Report | 1 | DOX CR (1) | None reported | 10 weeks |
| Knoell (2009) | Case Report | 1 | ADA CR (1) | None reported | 6 months |
| Yun et al. (2009) | Retrospective Study | 54 | Topical steroid CR (10); NR (3) Oral Steroid CR (6); NR (2) Dapsone CR (2); NR (3) HCQ CR (1); NR (2) PUVA CR (1); NR (1) Cyclosporine CR (1); NR (1) Isotretinoin CR (1); NR (1) | NA | NA |
| Chiu et al. (2008) | Case Report | 1 | nb-UVB CR (1) | None reported | NA |
| Dadban et al. (2008) | Case Report | 1 | GA lesions mostly disappeared after treating the underlying neoplasm | None reported | NA |
| Gass et al. (2008) | Case Report | 1 | HCQ + oral steroid CR (1) | Non reported | 14 months |
| Hinckley et al. (2008) | Case Series | 2 | GA lesions mostly disappeared (1) and not responded (1) after treating the underlying neoplasm | None reported | NA |
| Karsai et al. (2008) | Case Report | 1 | Nd:YAG laser CR (1) | Prolonged erythema | 3 treatment sessions |
| Baskan et al. (2007) | Case Report | 1 | Isotretinoin + topical pimecrolimus CR (1) | None reported | 4 months |
| Marzano et al. (2007) | Case Report | 1 | GA lesions completely disappeared after treating the underlying disease | None reported | NA |
| Batchelor et al. (2006) | Case Report | 1 | Bath PUVA CR (1) | None reported | 7.5 weeks (15 sessions) |
| Hall et al. (2006) | Case Series | 2 | Hydroxyurea CR (2) | None reported | 5 months (1); 3 months (1) |
| Asano et al. (2006) | Case Report | 1 | Etretinate CR (1) | Mild liver enzyme elevation | Six weeks |
| Kiremitci et al. (2006) | Case Report | 1 | Dapsone + topical steroid CR (1) | None reported | NA |
| Kluger et al. (2006) | Case Report | 1 | GA lesions completely disappeared after Ribavirine and Peg-IFN gamma were discotinued | None reported | NA |
| Ma et al. (2006) | Case Report | 1 | GA lesions completely disappeared after treating the underlying disease | None reported | NA |
| Sahin et al. (2006) | Case Report | 1 | Isotretinoin CR (1) | None reported | 3 months |
| Shimizu et al. (2006) | Case Report | 1 | GA lesions mostly disappeared after treating the underlying neoplasms | None reported | NA |
| Eberlein-König et al. (2005) | Retrospective study | 8 | Dimethylfumarate CR (3); PR (4); NR (1) | Diarrhea, nausea (6); dizziness (2); flushing (1) | 1 to 8 months (mean: 4.3 months) |
| Hertl et al. (2005) | Case Report | 1 | Infliximab PR (1) | None reported | 6 months |
| Pasmatzi et al. (2005) | Case Report | 1 | Isotretinoin CR (1) | Cheilitis, dryness of mucosae, and a moderate increase in cholesterol and triglyceride serum levels. | 6 months |
| Rigopoulos et al. (2005) | Case Report | 1 | Topical pimecrolimus PR (1) | None Reported | 3 months |
| Schnopp et al. (2005) | Prospective Study | 20 | UVA1 CR (10); PR (8); NR (2) | None Reported | NA |
| Cannistraci et al. (2005) | Case Series | 9 | HCQ CR (9) | NA | 4 months |
| Kovich et al. (2005) | Case Report | 1 | DOX + topical steroid PR (1) | Gastrointestinal side effects | 3 months |
| Goffe (2004) | Case Report | 1 | Efalizumab CR (1) | None reported | 3 months |
| Jain et al. (2004) | Case Series | 4 | Topical tacrolimus CR (2); PR (2) | None reported | 6 weeks |
| Choi et al. (2003) | Case Report | 1 | Topical steroid PR (1) | None reported | 1 month |
| Oz et al. (2003) | Case Report | 1 | Oral steroid CR (1) | None reported | NA |
| Arroyo (2003) | Case Report | 1 | Isotretinoin PR (1) | NA | NA |
| Buendía-Eisman et al. (2003) | Case Report | 1 | Isotretinoin CR (1) | None reported | 6 months |
| Guardiano et al. (2003) | Case Report | 1 | Oral vitamin E + zileuton PR (1) | None reported | NA |
| Rubegni et al. (2003) | Case Report | 1 | intramuscular defibrotide CR (1) | None reported | 7 months |
| Kreuter et al. (2002) | Case Report | 1 | Dimethyl fumarate and monoethyl fumarate CR (1) | Temporary lymphocytopenia | 3 months |
| Smith et al. (2002) | Case Series | 3 | Oral vitamin E + zileuton CR (3) | None reported | From 2 to 3 months |
| Adams et al. (2002) | Case Report | 1 | Isotretinoin CR (1) | None reported | 1 year |
| Antony et al. (2001) | Case Report | 1 | Clofazimine CR (1) | None reported | NA |
| Erkek et al. (2001) | Case Report | 1 | Isotretinoin PR (1) | None reported | NA |
| Schulze-Dirks et al. (2001) | Case Report | 1 | Dimethyl fumarate and monoethyl fumarate CR (1) | None reported | 6 weeks |
| Granel et al. (2000) | Case Report | 1 | GA lesions completely disappeared after treating the underlying disease | None reported | NA |
| Setterfield et al. (1999) | Case Report | 1 | PUVA CR (1) | None reported | 7 months |
| Langrock et al. (1998) | Case Report | 1 | Bath PUVA CR (1) | None reported | Approximately 2 months (30 sessions) |
| Szegedi et al. (1998) | Case Report | 1 | PUVA CR (1) | Post-inflammatory hyperpigmentation | 3 weeks |
| Wolf et al. (1998) | Case Report | 1 | Dapsone CR (1) | None reported | NA |
| Muchenberger et al. (1997) | Prospective Study | 4 | UVA1 CR (1); PR (3) | None reported | 3 weeks |
| Tang et al. (1996) | Case Report | 1 | Isotretinoin CR (1) | None reported | NA |
| Ho (1995) | Case Report | 2 | Cyclosporine PR (1); NR (1) | None reported | 3 months |
| Filotico et al. (1994) | Case Report | 1 | Cyclosporine CR (1) | None reported | 4 months |
| Smith et al. (1994) | Prospective Study | 10 | Potassium iodide CR (1); PR (3); NR (2) | Rhinorrhea (3); metallic taste (1); transient acneiform eruption (2); | 12 months with crossover at 6 months |
| Simon et al. (1994) | Prospective Study | 6 | Chloroquine CR (2)  HCQ CR (4) | None reported | NA |
| Rubel et al. (1993) | Case Report | 1 | PTX CR (1) | None reported | 4 months |
| Botella-Estrada et al. (1993) | Case Report | 1 | Dapsone NR (1)  Etretinate PR (1) | Etretinate: hair loss | Dapsone: 4 months  Etretinate: 7 months |
| Burg (1992) | Case Report | 1 | Topical Vitamin E PR (1) | None reported | 2 weeks |
| Schleicher et al. (1992) | Case Report | 1 | Isotretinoin CR (1) | Mild liver enzyme elevation | 10 weeks |
| McGregor et al. (1992) | Case Report | 1 | GA lesions completely disappeared after treating the underlying disease | None reported | NA |
| Vassileva et al. (1992) | Case Series | 2 | GA lesions completely disappeared after treating the underlying neoplasm (2) | None reported | NA |
| Kerker et al. (1990) | Prospective Study | 5 | PUVA CR (5) | Post-inflammatory hyperpigmentation | 4 to 20 months |
| Hindson et al. (1988) | Case Series | 3 | PUVA CR (1); PR (2) | None reported | 6 months to 1 year |
| Leenutaphong et al. (1988) | Case Report | 1 | GA lesions completely disappeared after treating the underlying disease | None reported | NA |
| Carlin et al. (1987) | Case Report | 1 | HCQ CR (1) | NA | NA |
| Willemsen et al. (1987) | Case Report | 1 | GA lesions completely disappeared after treating the underlying disease | None reported | NA |
| Czarnecki et al. (1986) | Case Series | 6 | Dapsone CR (6) | NA | From 2 to 20 months |
| Steiner et al. (1985) | Prospective Study | 16 | Dapsone CR (4); PR (3); NR (1); NE (2) | Headache and weakness (5) | Mean: 10.6 weeks |
| Saied et al. (1980) | Case Series | 2 | Dapsone CR (2) | Non reported | 10 weeks (1); 10 months (1) |
| CR: Complete Response; PR: Partial Response; NR: No Response; NE: Not Evaluated; NA: Not Available; BARI: Baricitinib; DUPI: Dupilumab; DOX: Doxycycline; HCQ: Hydroxychloroquine; ILK: Intralesional Kenalog; i.m. steroid: Intramuscular steroid; MMF: Mycophenolate mofetil; MTX: Methotrexate; ROM therapy: Rifampin, Ofloxacin, Minocycline; PTX: Pentoxifylline; TTO: Tea Tree Oil; UPA: Upadacitinib | | | | | |
