# Supplementary material for: Management Strategies for Generalised Granuloma Annulare: A Systematic Review of Current and Emerging Therapies
Source: Australas J Dermatol. 2025 Jun 30;66(6):329–37. doi: 10.1111/ajd.14560 (PMC12418146; doi:10.1111/ajd.14560)
Supplement: Supplementary file 3 — Data S1. [file AJD-66-329-s003.docx]

**Supplemental references**

1. Yang YW, Lehrer MD, Mangold AR, Yiannias JA, Nelson SA, Pittelkow MR. Treatment of granuloma annulare and related granulomatous diseases with sulphasalazine: a series of 16 cases. *J Eur Acad Dermatol Venereol*. 2021;35(1):211-215. doi:10.1111/jdv.16356
2. Megna M, Sidikov A, Ruggiero A, et al. A case of generalized granuloma annulare successfully treated by hydroxychloroquine. *Dermatol Ther*. 2020;33(6):e13894. doi:10.1111/dth.13894
3. Nordmann TM, Kim JR, Dummer R, Anzengruber F. A Monocentric, Retrospective Analysis of 61 Patients with Generalized Granuloma Annulare. *Dermatology*. 2020;236(4):369-374. doi:10.1159/000507247
4. King SA, Masood S, Youngberg GA, Brown E, Leicht SS. Generalized granuloma annulare heralding relapse of non-Hodgkin lymphoma. *JAAD Case Rep*. 2020;6(6):534-536. Published 2020 Apr 24. doi:10.1016/j.jdcr.2020.03.016
5. Durgin JS, Shields BE, Rosenbach M. Generalized granuloma annulare: A widespread response to limited application of compounded 2% topical tofacitinib. *JAAD Case Rep*. 2020;6(10):1113-1115. Published 2020 Aug 8. doi:10.1016/j.jdcr.2020.07.054
6. Blum S, Altman D. Treatment of generalized granuloma annulare with apremilast: A report of 2 cases. *JAAD Case Rep*. 2019;5(11):976-978. Published 2019 Oct 24. doi:10.1016/j.jdcr.2019.09.015
7. Clapé A, Vanhaecke C, Durlach A, et al. A Case of Generalized Interstitial Granuloma Annulare and Arthritis Associated with Breast Cancer. *Acta Derm Venereol*. 2019;99(2):244-245. doi:10.2340/00015555-3056
8. Torisu Y, Horai Y, Michitsuji T, et al. Giant Cell Arteritis with Generalized Granuloma Annulare. *Intern Med*. 2019;58(8):1173-1177. doi:10.2169/internalmedicine.1978-18
9. Xu Q, Gu Y, Li Y, Ling B, Yu H, Yao Z. Concurrence of generalized perforating and subcutaneous granuloma annulare in a 4-year-old boy with latent tuberculosis infection successfully treated with low-dose hydroxychloroquine. *J Dermatol*. 2020;47(2):e71-e72. doi:10.1111/1346-8138.15152
10. Bishnoi A, Raj D, Vinay K, Dogra S. Refractory Generalized Granuloma Annulare Treated With Oral Apremilast. *JAMA Dermatol*. 2019;155(11):1318-1320. doi:10.1001/jamadermatol.2019.2130
11. Chandan N, Boen M, Lake EP, Aronson I. Successful treatment of two individual cases of generalized granuloma annulare with amoxicillin/clavulanic acid and a combination of doxycycline and pentoxifylline. *Dermatol Online J*. 2018;24(8):13030/qt9161p8z0. Published 2018 Aug 15.
12. Naka F, Strober BE. Methotrexate treatment of generalized granuloma annulare: a retrospective case series. *J Dermatolog Treat*. 2018;29(7):720-724. doi:10.1080/09546634.2018.1447075
13. Nambiar KG, Jagadeesan S, Balasubramanian P, Thomas J. Successful Treatment of Generalized Granuloma Annulare with Pentoxifylline. *Indian Dermatol Online J*. 2017;8(3):218-220. doi:10.4103/2229-5178.206119
14. Javorsky E, Perkins A, Scharf MJ. Resolution of disseminated granuloma annulare with removal of surgical hardware. *Cutis*. 2016;98(1):E10-E11.
15. Cozzani E, Basso D, Cimmino MA, et al. Generalized annular granuloma associated with crowned dens syndrome, which resolved with colchicine treatment. *Clin Exp Dermatol*. 2016;41(6):640-642. doi:10.1111/ced.12863
16. Mikami E, Yanase M, Ito M, Kanzaki A, Saeki H. Generalized granuloma annulare successfully treated with narrowband ultraviolet B and anti-hepatitis C virus therapy. *J Dermatol*. 2016;43(8):975-977. doi:10.1111/1346-8138.13320
17. Pavlovsky M, Samuelov L, Sprecher E, Matz H. NB-UVB phototherapy for generalized granuloma annulare. *Dermatol Ther*. 2016;29(3):152-154. doi:10.1111/dth.12315
18. Yong A, Chong WS, Pan JY. Disseminated granuloma annulare responding to narrowband UVB phototherapy. *Photodermatol Photoimmunol Photomed*. 2016;32(2):107-109. doi:10.1111/phpp.12223
19. Errichetti E, Stinco G, Pegolo E, Patrone P. Generalized Granuloma Annulare in a Cirrhotic Patient Treated with Narrowband Ultraviolet B Therapy. *Indian J Dermatol*. 2016;61(1):127. doi:10.4103/0019-5154.174203
20. Min MS, Lebwohl M. Treatment of recalcitrant granuloma annulare (GA) with adalimumab: A single-center, observational study. *J Am Acad Dermatol*. 2016;74(1):127-133. doi:10.1016/j.jaad.2015.09.015
21. Bala HR, Khan S, Chong AH. Two cases of generalised granuloma annulare successfully treated with acitretin and NB UVB therapy. *Australas J Dermatol*. 2016;57(4):327-329. doi:10.1111/ajd.12500
22. Cunningham L, Kirby B, Lally A, Collins P. The efficacy of PUVA and narrowband UVB phototherapy in the management of generalised granuloma annulare. *J Dermatolog Treat*. 2016;27(2):136-139. doi:10.3109/09546634.2015.1087461
23. Garg S, Baveja S. Monthly rifampicin, ofloxacin, and minocycline therapy for generalized and localized granuloma annulare. *Indian J Dermatol Venereol Leprol*. 2015;81(1):35-39. doi:10.4103/0378-6323.148564
24. Mahmood T, Mansouri B, Menter A. Successful treatment of generalized granuloma annulare with adalimumab. *Clin Exp Dermatol*. 2015;40(5):537-539. doi:10.1111/ced.12598
25. Solano-López G, Concha-Garzón MJ, de Argila D, Daudén E. Successful Treatment of Disseminated Granuloma Annulare With Narrowband UV-B Phototherapy. *Actas Dermosifiliogr*. 2015;106(3):240-241. doi:10.1016/j.ad.2014.08.004
26. Acharya U. Successful treatment of disseminated granuloma annulare with oral fumaric acid esters. *Int J Dermatol*. 2013;52(5):633-634. doi:10.1111/j.1365-4632.2011.04930.x
27. Pătraşcu V, Giurcă C, Ciurea RN, Georgescu CV. Disseminated granuloma annulare: study on eight cases. *Rom J Morphol Embryol*. 2013;54(2):327-331.
28. Bégon E, Petitjean B, Bachmeyer C. Efficacité prolongée du méthotrexate dans un cas de granulome annulaire généralisé [Long-term efficacy of methotrexate in a patient with generalized granuloma annulare]. *Ann Dermatol Venereol*. 2013;140(2):136-137. doi:10.1016/j.annder.2012.11.004
29. Bronfenbrener R, Ragi J, Milgraum S. Granuloma annulare treated with excimer laser. *J Clin Aesthet Dermatol*. 2012;5(11):43-45.
30. Browne F, Turner D, Goulden V. Psoralen and ultraviolet A in the treatment of granuloma annulare. *Photodermatol Photoimmunol Photomed*. 2011;27(2):81-84. doi:10.1111/j.1600-0781.2011.00574.x
31. Dornelles SI, Poziomczyk CS, Boff A, Köche B, Dornelles Mde A, Richter GK. Generalized perforating granuloma annulare. *An Bras Dermatol*. 2011;86(2):327-331. doi:10.1590/s0365-05962011000200016
32. Jantke ME, Bertsch HP, Schön MP, Fuchs T. Therapie des Granuloma anulare disseminatum mit Anthralin [Treatment of disseminated granuloma annulare with anthralin]. *Hautarzt*. 2011;62(12):935-939. doi:10.1007/s00105-011-2185-y
33. Passeron T, Fusade T, Vabres P, et al. Treatment of granuloma annulare with the 595-nm pulsed dye laser, a multicentre retrospective study with long-term follow-up. *J Eur Acad Dermatol Venereol*. 2013;27(6):785-788. doi:10.1111/j.1468-3083.2011.04402.x
34. Wollina U, Langner D. Treatment of disseminated granuloma annulare recalcitrant to topical therapy: a retrospective 10-year analysis with comparison of photochemotherapy alone versus photochemotherapy plus oral fumaric acid esters. *J Eur Acad Dermatol Venereol*. 2012;26(10):1319-1321. doi:10.1111/j.1468-3083.2011.04320.x
35. Bhushan P, Aggarwal A, Yadav R, Baliyan V. Generalized granuloma annulare with open comedones in photoexposed areas. *Clin Exp Dermatol*. 2011;36(5):495-498. doi:10.1111/j.1365-2230.2011.04025.x
36. Nagase K, Koba S, Okawa T, Inoue T, Misago N, Narisawa Y. Generalized granuloma annulare following BCG vaccination, mimicking papular tuberculid. *Eur J Dermatol*. 2011;21(6):1001-1002. doi:10.1684/ejd.2011.1512
37. Torres T, Pinto Almeida T, Alves R, Sanches M, Selores M. Treatment of recalcitrant generalized granuloma annulare with adalimumab. *J Drugs Dermatol*. 2011;10(12):1466-1468.
38. Mazzatenta C, Ghilardi A, Grazzini M. Treatment of disseminated granuloma annulare with allopurinol: case report. *Dermatol Ther*. 2010;23 Suppl 1:S24-S27. doi:10.1111/j.1529-8019.2009.01283.x
39. Milicić V, Ravić-Nikolić A, Jovović-Dagović B, Ristić G, Mitrović S. Generalized granuloma annulare presenting as arcuate dermal erythema. *Acta Dermatovenerol Alp Pannonica Adriat*. 2010;19(1):25-27.
40. Murdaca G, Colombo BM, Barabino G, Caiti M, Cagnati P, Puppo F. Anti-tumor necrosis factor-α treatment with infliximab for disseminated granuloma annulare. *Am J Clin Dermatol*. 2010;11(6):437-439. doi:10.2165/11311040-000000000-00000
41. Ine K, Kabashima K, Koga C, Kobayashi M, Tokura Y, Kabashima K. Eruptive generalized granuloma annulare presenting with numerous micropapules. *Int J Dermatol*. 2010;49(1):104-105. doi:10.1111/j.1365-4632.2008.03661.x
42. Werchau S, Enk A, Hartmann M. Generalized interstitial granuloma annulare--response to adalimumab. *Int J Dermatol*. 2010;49(4):457-460. doi:10.1111/j.1365-4632.2010.04348.x
43. Marcus DV, Mahmoud BH, Hamzavi IH. Granuloma annulare treated with rifampin, ofloxacin, and minocycline combination therapy. *Arch Dermatol*. 2009;145(7):787-789. doi:10.1001/archdermatol.2009.55
44. Piaserico S, Zattra E, Linder D, Peserico A. Generalized granuloma annulare treated with methylaminolevulinate photodynamic therapy. *Dermatology*. 2009;218(3):282-284. doi:10.1159/000195675
45. Weber HO, Borelli C, Röcken M, Schaller M. Treatment of disseminated granuloma annulare with low-dose fumaric acid. *Acta Derm Venereol*. 2009;89(3):295-298. doi:10.2340/00015555-0647
46. Aşkin U, Durdu M, Senel E. Generalized granuloma annulare in a patient with myelocytic leukemia and chronic hepatitis B virus infection. *Indian J Dermatol Venereol Leprol*. 2009;75(3):287-289. doi:10.4103/0378-6323.51251
47. Duarte AF, Mota A, Pereira MA, Baudrier T, Azevedo F. Generalized granuloma annulare--response to doxycycline. *J Eur Acad Dermatol Venereol*. 2009;23(1):84-85. doi:10.1111/j.1468-3083.2008.02707.x
48. Knoell KA. Efficacy of adalimumab in the treatment of generalized granuloma annulare in monozygotic twins carrying the 8.1 ancestral haplotype. *Arch Dermatol*. 2009;145(5):610-611. doi:10.1001/archdermatol.2009.92
49. Yun JH, Lee JY, Kim MK, et al. Clinical and pathological features of generalized granuloma annulare with their correlation: a retrospective multicenter study in Korea. *Ann Dermatol*. 2009;21(2):113-119. doi:10.5021/ad.2009.21.2.113
50. Chiu ML, Tang MB. Generalized granuloma annulare associated with gastrointestinal stromal tumour: case report and review of clinical features and management. *Clin Exp Dermatol*. 2008;33(4):469-471. doi:10.1111/j.1365-2230.2007.02681.x
51. Dadban A, Slama B, Azzedine A, Lepeu G. Widespread granuloma annulare and Hodgkin's disease. *Clin Exp Dermatol*. 2008;33(4):465-468. doi:10.1111/j.1365-2230.2008.02730.x
52. Gass JK, Todd PM, Rytina E. Generalized granuloma annulare in a photosensitive distribution resolving with scarring and milia formation. *Clin Exp Dermatol*. 2009;34(5):e53-e55. doi:10.1111/j.1365-2230.2008.03183.x
53. Hinckley MR, Walsh SN, Molnár I, Sheehan DJ, Sangueza OP, Yosipovitch G. Generalized granuloma annulare as an initial manifestation of chronic myelomonocytic leukemia: a report of 2 cases. *Am J Dermatopathol*. 2008;30(3):274-277. doi:10.1097/DAD.0b013e318166ea1a
54. Karsai S, Hammes S, Rütten A, Raulin C. Fractional photothermolysis for the treatment of granuloma annulare: a case report. *Lasers Surg Med*. 2008;40(5):319-322. doi:10.1002/lsm.20640
55. Baskan EB, Turan A, Tunali S. A case of generalized granuloma annulare with myelodysplastic syndrome: successful treatment with systemic isotretinoin and topical pimecrolimus 1% cream combination. *J Eur Acad Dermatol Venereol*. 2007;21(5):693-695. doi:10.1111/j.1468-3083.2006.01989.x
56. Marzano AV, Ramoni S, Alessi E, Caputo R. Generalized granuloma annulare and eruptive folliculitis in an HIV-positive man: resolution after antiretroviral therapy. *J Eur Acad Dermatol Venereol*. 2007;21(8):1114-1116. doi:10.1111/j.1468-3083.2006.02103.x
57. Batchelor R, Clark S. Clearance of generalized papular umbilicated granuloma annulare in a child with bath PUVA therapy. *Pediatr Dermatol*. 2006;23(1):72-74. doi:10.1111/j.1525-1470.2006.00175.x
58. Hall CS, Zone JJ, Hull CM. Treatment of recalcitrant disseminated granuloma annulare with hydroxyurea. *J Am Acad Dermatol*. 2008;58(3):525. doi:10.1016/j.jaad.2006.08.054
59. Asano Y, Saito A, Idezuki T, Igarashi A. Generalized granuloma annulare treated with short-term administration of etretinate. *J Am Acad Dermatol*. 2006;54(5 Suppl):S245-S247. doi:10.1016/j.jaad.2006.01.028
60. Kiremitci U, Karagulle S, Topcu E, et al. Generalized granuloma annulare resolving to anetoderma. *Dermatol Online J*. 2006;12(7):16. Published 2006 Dec 10.
61. Kluger N, Moguelet P, Chaslin-Ferbus D, Khosrotherani K, Aractingi S. Generalized interstitial granuloma annulare induced by pegylated interferon-alpha. *Dermatology*. 2006;213(3):248-249. doi:10.1159/000095048
62. Ma HJ, Zhu WY, Yue XZ. Generalized granuloma annulare associated with chronic hepatitis B virus infection. *J Eur Acad Dermatol Venereol*. 2006;20(2):186-189. doi:10.1111/j.1468-3083.2005.01366.x
63. Sahin MT, Türel-Ermertcan A, Oztürkcan S, Türkdogan P. Generalized granuloma annulare in a patient with type II diabetes mellitus: successful treatment with isotretinoin. *J Eur Acad Dermatol Venereol*. 2006;20(1):111-114. doi:10.1111/j.1468-3083.2006.01337.x
64. Shimizu S, Yasui C, Tsuchiya K. Atypical generalized granuloma annulare associated with two visceral cancers. *J Am Acad Dermatol*. 2006;54(5 Suppl):S236-S238. doi:10.1016/j.jaad.2005.10.045
65. Eberlein-König B, Mempel M, Stahlecker J, Forer I, Ring J, Abeck D. Disseminated granuloma annulare--treatment with fumaric acid esters. *Dermatology*. 2005;210(3):223-226. doi:10.1159/000083514
66. Hertl MS, Haendle I, Schuler G, Hertl M. Rapid improvement of recalcitrant disseminated granuloma annulare upon treatment with the tumour necrosis factor-alpha inhibitor, infliximab. *Br J Dermatol*. 2005;152(3):552-555. doi:10.1111/j.1365-2133.2005.06371.x
67. Pasmatzi E, Georgiou S, Monastirli A, Tsambaos D. Temporary remission of disseminated granuloma annulare under oral isotretinoin therapy. *Int J Dermatol*. 2005;44(2):169-171. doi:10.1111/j.1365-4632.2005.02229.x
68. Rigopoulos D, Prantsidis A, Christofidou E, Ioannides D, Gregoriou S, Katsambas A. Pimecrolimus 1% cream in the treatment of disseminated granuloma annulare. *Br J Dermatol*. 2005;152(6):1364-1365. doi:10.1111/j.1365-2133.2005.06594.x
69. Schnopp C, Tzaneva S, Mempel M, Schulmeister K, Abeck D, Tanew A. UVA1 phototherapy for disseminated granuloma annulare. *Photodermatol Photoimmunol Photomed*. 2005;21(2):68-71. doi:10.1111/j.1600-0781.2005.00145.x
70. Cannistraci C, Lesnoni La Parola I, Falchi M, Picardo M. Treatment of generalized granuloma annulare with hydroxychloroquine. *Dermatology*. 2005;211(2):167-168. doi:10.1159/000086452
71. Kovich O, Burgin S. Generalized granuloma annulare. *Dermatol Online J*. 2005;11(4):23. Published 2005 Dec 30.
72. Goffe BS. Disseminated granuloma annulare resolved with the T-cell modulator efalizumab. *Arch Dermatol*. 2004;140(10):1287-1288. doi:10.1001/archderm.140.10.1287
73. Jain S, Stephens CJ. Successful treatment of disseminated granuloma annulare with topical tacrolimus. *Br J Dermatol*. 2004;150(5):1042-1043. doi:10.1111/j.1365-2133.2004.05947.x
74. Choi JC, Bae JY, Cho S, Choi JH, Sung KJ, Moon KC, et al. Generalized perforating granuloma annulare in an infant. *Pediatr Dermatol*. 2003;20(2):131-133. doi:10.1046/j.1525-1470.2003.20207.x
75. Oz O, Tursen U, Yildirim O, Kaya TI, Ikizoglu G. Uveitis associated with granuloma annulare. *Eur J Ophthalmol*. 2003;13(1):93-95. doi:10.1177/112067210301300116
76. Arroyo MP. Generalized granuloma annulare. *Dermatol Online J*. 2003;9(4):13.
77. Buendía-Eisman A, Ruiz-Villaverde R, Blasco-Melguizo J, Serrano-Ortega S. Generalized annular granuloma: response to isotretinoin. *Int J Dermatol*. 2003;42(4):321-322. doi:10.1046/j.1365-4362.2003.01799_4.x
78. Guardiano RA, Lee W, Norwood C, Darling T. Generalized granuloma annulare in a patient with adult onset diabetes mellitus. *J Drugs Dermatol*. 2003;2(6):666-668.
79. Rubegni P, Sbano P, Fimiani M. A case of disseminated granuloma annulare treated with defibrotide: complete clinical remission and progressive hair darkening. *Br J Dermatol*. 2003;149(2):437-439. doi:10.1046/j.1365-2133.2003.05462.x
80. Kreuter A, Gambichler T, Altmeyer P, Brockmeyer NH. Treatment of disseminated granuloma annulare with fumaric acid esters. *BMC Dermatol*. 2002;2:5. Published 2002 Mar 19. doi:10.1186/1471-5945-2-5
81. Smith KJ, Norwood C, Skelton H. Treatment of disseminated granuloma annulare with a 5-lipoxygenase inhibitor and vitamin E. *Br J Dermatol*. 2002;146(4):667-670. doi:10.1046/j.1365-2133.2002.04590.x
82. Adams DC, Hogan DJ. Improvement of chronic generalized granuloma annulare with isotretinoin. *Arch Dermatol*. 2002;138(11):1518-1519. doi:10.1001/archderm.138.11.1518
83. Antony F, Holden CA. Sweet's syndrome in association with generalized granuloma annulare in a patient with previous breast carcinoma. *Clin Exp Dermatol*. 2001;26(8):668-670. doi:10.1046/j.1365-2230.2001.00914.x
84. Erkek E, Karaduman A, Bükülmez G, Sentürk N, Ozkaya O. An unusual form of generalized granuloma annulare in a patient with insulin-dependent diabetes mellitus. *Acta Derm Venereol*. 2001;81(1):48-50. doi:10.1080/00015550121061
85. Schulze-Dirks A, Petzoldt D. Granuloma anulare disseminatum--erfolgreiche Therapie mit Fumarsäureester [Granuloma annulare disseminatum: successful therapy with fumaric acid ester]. *Hautarzt*. 2001;52(3):228-230. doi:10.1007/s001050051294
86. Granel B, Serratrice J, Rey J, et al. Chronic hepatitis C virus infection associated with a generalized granuloma annulare. *J Am Acad Dermatol*. 2000;43(5 Pt 2):918-919
87. Setterfield J, Huilgol SC, Black MM. Generalised granuloma annulare successfully treated with PUVA. *Clin Exp Dermatol*. 1999;24(6):458-460. doi:10.1046/j.1365-2230.1999.00532.x
88. Langrock A, Weyers W, Schill WB. Balneophotochemotherapie bei disseminiertem Granuloma anulare [Balneophotochemotherapy in disseminated granuloma annulare]. *Hautarzt*. 1998;49(4):303-306. doi:10.1007/s001050050745
89. Szegedi A, Bégány A, Hunyadi J. Successful treatment of generalized granuloma annulare with polyethylene sheet bath PUVA. *Acta Derm Venereol*. 1999;79(1):84-85. doi:10.1080/000155599750011813
90. Wolf F, Grezard P, Berard F, Clavel G, Perrot H. Generalized granuloma annulare and hepatitis B vaccination. *Eur J Dermatol*. 1998;8(6):435-436.
91. Muchenberger S, Schöpf E, Simon JC. Phototherapy with UV-A-I for generalized granuloma annulare. *Arch Dermatol*. 1997;133(12):1605. doi:10.1001/archderm.1997.03890480131025
92. Tang WY, Chong LY, Lo KK. Resolution of generalized granuloma annulare with isotretinoin therapy. *Int J Dermatol*. 1996;35(6):455-456. doi:10.1111/j.1365-4362.1996.tb03034.x
93. Ho VC. Cyclosporine in the treatment of generalized granuloma annulare. *J Am Acad Dermatol*. 1995;32(2 Pt 1):298. doi:10.1016/0190-9622(95)90159-0
94. Filotico R, Vena GA, Coviello C, Angelini G. Cyclosporine in the treatment of generalized granuloma annulare. *J Am Acad Dermatol*. 1994;30(3):487-488. doi:10.1016/s0190-9622(08)81946-9
95. Smith JB, Hansen CD, Zone JJ. Potassium iodide in the treatment of disseminated granuloma annulare. *J Am Acad Dermatol*. 1994;30(5 Pt 1):791-792. doi:10.1016/s0190-9622(08)81516-2
96. Simon M Jr, von den Driesch P. Antimalarials for control of disseminated granuloma annulare in children. *J Am Acad Dermatol*. 1994;31(6):1064-1065. doi:10.1016/s0190-9622(09)80089-3
97. Rubel DM, Wood G, Rosen R, Jopp-McKay A. Generalised granuloma annulare successfully treated with pentoxifylline. *Australas J Dermatol*. 1993;34(3):103-108. doi:10.1111/j.1440-0960.1993.tb00875.x
98. Botella-Estrada R, Guillen C, Sanmartin O, Aliaga A. Disseminated granuloma annulare: resolution with etretinate therapy. *J Am Acad Dermatol*. 1992;26(5 Pt 1):777-778. doi:10.1016/s0190-9622(08)80559-2
99. Burg G. Disseminated granuloma anulare: therapy with vitamin E topically. *Dermatology*. 1992;184(4):308-309. doi:10.1159/000247580
100. Schleicher SM, Milstein HJ, Lim SJ, Stanton CD. Resolution of disseminated granuloma annulare with isotretinoin. *Int J Dermatol*. 1992;31(5):371-372. doi:10.1111/j.1365-4362.1992.tb03970.x
101. McGregor JM, McGibbon DH. Disseminated granuloma annulare as a presentation of acquired immunodeficiency syndrome (AIDS). *Clin Exp Dermatol*. 1992;17(1):60-62. doi:10.1111/j.1365-2230.1992.tb02538.x
102. Vassileva S, Krasteva M, Marina S, Tsankov N. Widespread granuloma annulare and cervical adenocarcinoma. *Int J Dermatol*. 1992;31(11):819. doi:10.1111/j.1365-4362.1992.tb04255.x
103. Kerker BJ, Huang CP, Morison WL. Photochemotherapy of generalized granuloma annulare. *Arch Dermatol*. 1990;126(3):359-361.
104. Hindson TC, Spiro JG, Cochrane H. PUVA therapy of diffuse granuloma annulare. *Clin Exp Dermatol*. 1988;13(1):26-27. doi:10.1111/j.1365-2230.1988.tb00644.x
105. Leenutaphong V, Hölzle E, Erckenbrecht J, Zuleger S, Plewig G. Remission of human immunodeficiency virus-associated generalized granuloma annulare under zidovudine therapy. *J Am Acad Dermatol*. 1988;19(6):1126-1127. doi:10.1016/s0190-9622(98)80014-5
106. Carlin MC, Ratz JL. A case of generalized granuloma annulare responding to hydroxychloroquine. *Cleve Clin J Med*. 1987;54(3):229-232. doi:10.3949/ccjm.54.3.229
107. Willemsen MJ, de Coninck AL, Jonckheer MH, Roseeuw DI. Autoimmune thyroiditis and generalized granuloma annulare: remission of the skin lesions after thyroxine therapy. *Dermatologica*. 1987;175(5):239-243. doi:10.1159/000248911
108. Czarnecki DB, Gin D. The response of generalized granuloma annulare to dapsone. *Acta Derm Venereol*. 1986;66(1):82-84.
109. Steiner A, Pehamberger H, Wolff K. Sulfone treatment of granuloma annulare. *J Am Acad Dermatol*. 1985;13(6):1004-1008. doi:10.1016/s0190-9622(85)70253-8
110. Saied N, Schwartz RA, Estes SA. Treatment of generalized granuloma annulare with dapsone. *Arch Dermatol*. 1980;116(12):1345-1346.
111. Fayyazi A, Schweyer S, Eichmeyer B, Herms J, Hemmerlein B, Radzun HJ et al. Expression of IFNgamma, coexpression of TNFalpha and matrix metalloproteinases and apoptosis of T lymphocytes and macrophages in granuloma annulare. *Arch Dermatol Res*. 2000;292(8):384-390. doi:10.1007/s004030000150
